# Mobility and migration in Byzantium: who gets to tell the story?

**DOI:** 10.1111/emed.12641

**Published:** 2023-06-22

**Authors:** Claudia Rapp

**Affiliations:** ^1^ University of Vienna

## Abstract

This article underlines the importance of approaching written sources for what they are: authorial constructs. This is true also for depictions of mobility and migration. Byzantine authors instrumentalized these for their own purposes beyond the event at hand. Authorial focus, along with the requirements of the chosen literary genre, is also the reason for the different scales of actors that appear in these texts, whether large blurry masses of nameless people, smaller groups with a distinct profile, or finely drawn individuals.

## Introduction

Byzantium offers rich material for the study of connectivity and interaction with other regions on multiple levels – economic, cultural, demographic. The geographic location of Byzantium lies at the intersection of the continents of Europe and Asia. Its capital Constantinople (modern Istanbul) was connected by sea through the Mediterranean with the regions to the west and through the Black Sea and the connected river routes with the regions to the north and east. A well‐established road system allowed traffic by land. Since Roman times, the Via Egnatia led through the Balkans to Italy, while several branches of the anachronistically called ‘Silk Road’ linked all the way up to China.

Recent publications have explored this aspect of transregional studies in a comparative perspective and helped to put Byzantium on the map as a major player in the history of the global Middle Ages, for example in the volume *Migration Histories of the Medieval Afroeurasian Transition Zone* or in the publication of conference papers on *Global Byzantium*. 1 But when it comes to interactions with other regions, Byzantium was not unique. Catherine Holmes and Naomi Standen, who led the research project ‘Defining the Global Middle Ages’ (2012–15), emphasize the ubiquity of this phenomenon: ‘Mobility was not only the province of particular types of medieval people (migrants, nomads, rulers, merchants or pilgrims) undertaking specific, often elite‐focused, activities but … a phenomenon that played a crucial structural and structuring role in almost every area of life, including resource‐gathering, politics, religious practices, “and perhaps, in the end, everything”.’ 2

At the same time, we must be wary of the impulse to generalize the positive effects of mobility. The mere presence of a distinct cultural group within another culture does not automatically result in mutual understanding, trust, or even adaptation and assimilation of the former to the latter, as Francesca Trivellato has shown with regard to the Sephardic merchants of Livorno in the seventeenth and eighteenth centuries, whose trading activities extended from the Atlantic to India. Pursuing ‘global history on a small scale’, the evidence she was able to assemble ‘challenges the assumption that the ability to lend money and delegate decisions to strangers was naturally coupled with the dissolution of corporate boundaries, the rise of individualism, and more tolerant attitudes’. 3

Approaching mobility with a greater degree of granularity helps to avoid the pitfalls of naive generalizations, even as the focus on one individual and her or his movements makes it possible to create a bigger picture of connectivities and entanglements. The extensive ‘shopping list’ of precious and mundane trading items created by Abraham, a Muslim trader from North Africa, preserved among the Cairo Genizah documents, opens up new vistas on the maritime connections between the continents of Africa, Europe and India in the twelfth century, as Elizabeth Lambourn has demonstrated. 4 The fate of Tamta Mqargrdzeli, the daughter of a Georgian military commander and a token in the hands of different rulers who laid claim to power in Anatolia in the thirteenth century, becomes – in the re‐telling of Antony Eastmond – a history of entanglements that extends from the Mediterranean to Central Asia. 5

The sources that undergird such investigations can be called ‘moving stories’, in the felicitous term coined by Jean‐Paul Ghobrial. In a model study, he traced the life of Joseph Georgirenes, archbishop of Samos, through documents in England, Spain and Argentina. Fleeing the Ottomans, the cleric arrived in London in 1676. He later was active in Spain and from there travelled to Buenos Aires, where court records document his presence in the 1690s. Along the way, those whom he encountered and especially those who questioned his religious credentials (Was he Orthodox? Was he Catholic?) and legitimacy, projected different identities onto him. 6 It is precisely in the context of dislocation that such identities are continually adapted and reshaped. Such historical case studies open exciting possibilities for a better understanding of the conjunction of mobility with the identity politics of others, as well as the identification strategies of the actors themselves and those whom they encounter along their life journey.

The present study highlights ‘moving stories’, to borrow Ghobrial’s term, in order to draw attention to the possibilities but also the interpretive challenges of studying mobility on the basis of written texts, with their own story‐telling impetus. The focus, in keeping with the profile of *Early Medieval Europe*, is on the period prior to the year 1000, with occasional forays into later centuries.

Mobility was common in Byzantium, as in all other societies. At the time of its emergence as the successor to the Roman Empire in the east, the empire we now call Byzantine encompassed 1.4 million square kilometres, with an estimated 28 million inhabitants, assuming a population density of 20 people per square kilometre. 7 Approximately 80 per cent worked in agriculture, but in the urban centres, first and foremost Constantinople and Thessaloniki, there were also artisans, traders and bureaucrats in charge of the imperial and ecclesiastical administration. These highly skilled people of the middle and upper strata of society often covered great distances in pursuit of their work. Those on the lower rungs of society, by contrast, were forced to relocate either by adverse circumstances or indeed compelled to do so by imperial decree.

This migratory aspect of Byzantine history has been addressed by specialists since the last decades of the past century. 8 Their studies tended to be based on a limited range of sources, mostly historical works written by elite authors that represent the view from the centre of power, supplemented by hagiographies composed by religious men that reflect their interest in the individual men and women of provincial society.

This article builds upon these lines of enquiry. It aims to investigate how we can gain a better understanding of the phenomenon of people on the move as they are reported in the Byzantine sources. It begins by assessing the usefulness of the terms ‘migration’ and ‘mobility’, and advocates the consideration of ‘personal agency’ as an essential component in any enquiry that wants to do justice to the role of the actors themselves in these historical processes. The second part discusses the Byzantine authors who report such events and their intentions in doing so. Particular emphasis is placed on issues of scale, i.e. the question of how the mobility of individuals and smaller or larger groups becomes a topic in different literary genres. In this way, this article hopes to contribute not only to the study of mobility and migration as historical phenomena, but specifically to a more nuanced understanding of why we know what we know, and why there is so much that still remains unknown.

## Looking for the sources

Since 2015, I have been fortunate to have pursued this line of enquiry – with a multi‐generational team of scholars and in frequent dialogue with historians of modern migrations – with the generous support of the Wittgenstein‐Award of the Austrian Science Fund. As the project title ‘Mobility, Microstructures and Personal Agency’ indicates, we also include the social dimension in this enquiry. 9 We are never dealing with abstract arrows on a map, but with individual men and women (and children, too) who have agency, who make decisions before, during and after their movement, and whose fate often depends on their social networks. Hence our emphasis on microstructures in conjunction with mobility. 10

Together, we have created a sourcebook for scholars and for classroom use, in the hope of fostering further work on this range of issues: *Mobility and Migration in Byzantium: A Sourcebook*, from which most of the following examples are taken. 11 The book covers the seventh to the fifteenth century. The starting point was chosen so as to facilitate collaboration with the Tübingen project group around Mischa Meier ‘Migration and Mobility in Late Antiquity and the Early Middle Ages’, who will be preparing a companion volume that covers the earlier centuries and includes Latin texts. Our great challenge was not only to ensure an even distribution of texts across the centuries, but especially to demonstrate that mobility is addressed across all manifestations of the written record of Byzantium.

Once we began looking, we found poems where the author assumes the position of the poetic ‘I’ to describe his eviction from his home and his joy when the emperor allows him to move back. At the end of the eleventh century, John Mauropous’ Poem 47 *On His Own Home When He Sold and Left It* begins in a mournful mood: ‘Do not be angry with me, my house, as having been left deserted and empty.’ The corresponding Poem 48 *When He Recovered His House* strikes a joyous note: ‘I have you back and look at you, my most beloved.’ 12 Court records that are preserved as part of the letter collection of Demetrios Chomatenos, archbishop of Ohrid (1216–36), mention a woman who undertook a long and arduous journey by sea and by land in order to secure her property rights:

Since she has an eager and manly spirit, Maria the daughter of Opsikianos began her journey in Kerkyra. Without concern for a woman’s weakness, she took upon herself the trouble of crossing the sea and covering long distances by land, in order to come to us, to appear before our own mediocrity and to bring forth her case. For this reason, her case was given a hearing today by our mediocrity in public and in addition to her accusation, she received our judgment (as follows). 13

Saints’ *Lives* describe the desperation of farmers driven to abandon their plots after droughts and famines: Saint Philaretos the Merciful, a provincial magnate whose *Vita* was composed in the early ninth century, provided generous assistance to a farmer who was in great despair after the sudden death of his ox, invoking God:

Lord, I had nothing but this yoke and yet you deprived me of this too. With what shall I feed my wife and my nine small children? How shall I pay taxes to the emperor? With what shall I pay my debts? Lord, you know that the ox that died had been bought on credit, and I am at a loss what to do. Therefore I shall leave home and run away to a far country before my creditors find out and fall upon me like wild beasts. 14

A displaced aristocrat reflects in his testament how he moved and settled in a new environment: Eustathios Boilas was forced ‘by a cruel fate’ to move with his household from Cappadocia to the eastern border regions with Armenia, where through hard work he succeeded in establishing himself and seeing his daughters make advantageous marriages to local grandees. 15 In a foundation charter for his new monastery, Gregory Pakourianos, a nobleman who proudly declares his Georgian descent, expressed his relief at settling down in what is now Bulgaria after decades on military campaign in the service of the Byzantine emperor, but stipulated that only Georgians should be allowed to join this community, in a striking display of resistance to assimilation. 16

The earliest Byzantine prayer book made for the use of priests (*euchologion*), copied in the late eighth century in southern Italy (Vatican, Biblioteca Apotolica Vaticana, Barb. gr. 336), contains a prayer for travel, suitable also for those going on pilgrimage, that asks for protection from brigands, robbers and inclement weather.

Prayer for those who are about to travel. God, our God, who was the travelling companion of your attendant Jacob, and who went abroad with your servant Joseph: be a companion along the way of this your servant [name to be inserted], o Lord, protect him from brigands, robbers and all stormy weather and return him in peace and strength, him who has observed all justice according to your commandments. And deem him worthy to return filled with all your earthly and heavenly goods. For the kingdom and the power and the glory are yours, Father [Son and Holy Spirit, for ever and ever. Amen.] 17

These texts do not make mobility their declared focus, but offer valuable information about it nonetheless. It is essential to cast a wide net in order to gather all relevant evidence even in sources where one would not necessarily expect to find information on mobility, otherwise we run the risk of overlooking a phenomenon that was so common that it only attracted the authors’ focused attention in special circumstances.

## Part One: migration and mobility

Migrations and the violent disruptions they caused have become a structuring principle for the study of the history of Byzantium: the arrival of the Arabs in the middle of the seventh century brought the end of the early Byzantine period and severed the unity of the Mediterranean as the Roman *mare nostrum*; the Crusaders’ capture of Constantinople in 1204 marked the end of the middle Byzantine period; and the Ottoman takeover of the capital city in 1453 spelled the end of the Byzantine Empire. 18 These large movements of people from outside brought warfare and destruction and threatened the very existence of the empire. Classified as ‘invasions’, they are often visualized on historical maps with arrows that penetrate an empire delineated by sharp borders that are, by implication, imagined as inviolable, intact and uniform. Often, the metaphorical language of the forces of nature is invoked to describe such movements as occurring in ‘waves’.

Migration *within* the Byzantine Empire (*Binnenmigration*) has also been the subject of study. It could occur as a result of an imperial policy of forced resettlement or be the chosen path of religious and ethnic groups acting on their own account, although often under duress. 19 The Armenians represent a special and much‐studied case. Escaping regional and dynastic struggles, Armenian nobles and their families found safety and employment in Byzantium. The fact that many people of Armenian descent achieved high positions in the administration, and even the imperial throne in the case of Leo V ‘the Armenian’ (r. 813–20), made them a success story of resourcefulness and social advancement. 20 The laudatory biography of Emperor Basil I (r. 867–86), composed at the behest of his grandson Constantine VII, proudly traced his ancestry to the royal house of the Arsacids in fifth‐century Armenia, whence his forebears had come to Constantinople for refuge. Subsequent emperors moved the clan and their descendants to Macedonia – indeed, Basil was known to his contemporaries as ‘the Macedonian’ – where they had to relocate three times, reaching their final destination in Adrianople under Emperor Heraclius (r. 610–41).

Finding that that place suited them well, they banded together into a clan and tribe of their own, as it were; multiplied, and became quite prosperous. They <also> preserved the purity of their ancestral stock by keeping it free of any admixture. 21

This passage illustrates how migrations bring identities into sharp focus, as the migrants are said to fear that the adaptation to their new surroundings would come at the expense of abandoning their accustomed mode of being. Emphasizing this aspect was a useful narrative strategy to extol the purity of Basil’s royal ancestry, given the fact that he was actually an upstart from the provinces who gained the throne in Constantinople through a combination of craftiness, connections and good luck. 22 In reality, there is plenty of evidence, especially for the sixth to eleventh century, that Armenians who achieved high military and administrative positions and married into Byzantine society assimilated to the extent that their descendants did not choose to present themselves as ‘Armenian’. 23

Large‐scale migration within the Byzantine Empire could occur for manifold reasons, and was not always voluntary. Persecutions played a major role. The religious sect of the Paulicians in the ninth century was expelled from Asia Minor to Syria, southern Italy and the Balkans. 24 Forced resettlements as part of imperial policy are reported with unsettling frequency. Especially during the active confrontation with the Arabs, from the seventh to the ninth century, Byzantine emperors employed a policy of forced migration (*Zwangsmigration*) in order to ensure an even distribution of settlements in those regions where warfare had resulted in depopulation. Given the large number of such forcible movements, it is surprising that no sources survive that speak to the administrative and organizational processes that would have been necessary in their preparation and execution.

Yannis Stouraitis has analysed the different reasons for migrations in Byzantium, distinguishing between voluntary and involuntary migrations, and establishing further subcategories (Table 1). 25 As Stouraitis would be the first to admit, however, such a diagram has its limitations and can never do justice to the complex life situations of the individuals involved. Indeed, economic reasons are featured under multiple headings, pointing to the fact that it is often impossible to distinguish whether the decision to relocate in search of a better life, whether after a natural disaster or under fiscal duress, was voluntary or involuntary.

**Table 1:**
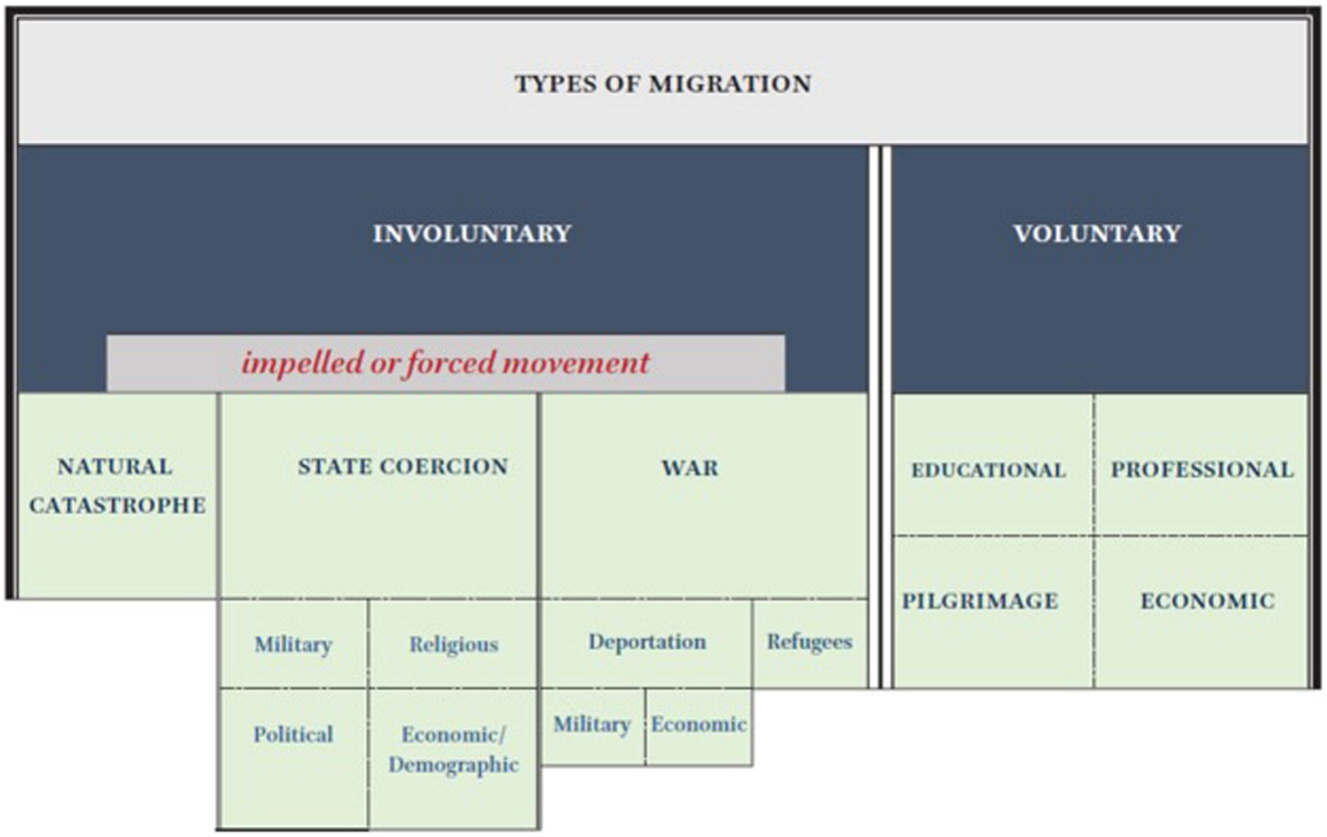
Yannis Stouraitis, types of migration

### Scale and its representation in the texts

What Stouraitis’ diagram also does not show is the issue of scale. Depending on its impetus and reason, mobility affected different numbers of people. Involuntary migration as a result of warfare or due to imperially ordained forced resettlement resulted in the movement of large numbers of people from the same region, while voluntary migration that was necessitated by one’s station in life only concerned an individual and possibly her or his family or household. People often moved for professional reasons. Imperial or elite women (often at a very young age) became brides to husbands in distant regions and moved there with their own entourage. 26 Relocation was required of bishops, metropolitans or patriarchs when they were appointed to a new see. Rising through the ranks of the military meant a lifetime spent in training or on campaign. 27 Acquiring the high level of education that was the prerequisite for an advantageous position at the court, in the imperial administration or in the higher clergy, necessitated a move to the large urban centres, Constantinople, but also Thessaloniki, and in the early Byzantine period also Alexandria, Antioch, Athens or Berytus. A very wide range of sources – documentary, legal, even liturgical, and narrative – mention the movement of individuals, but often they do so only in passing, while archaeology and material culture offer tangible evidence for the movement of objects that were carried across long distances by their owners, or brought by traders and diplomats. 28

The issue of scale also affects the way that movement is perceived and expressed by Byzantine authors and the modern scholars who depend on them. When large groups of people labelled with ethnonyms (the Armenians) or compartmentalized as heretics (the Paulicians) are described by Byzantine historians as moving from one region to another, scholars commonly identify this as ‘migration’. 29 The actors in these large‐scale movements, even if they occur under peaceable conditions, remain nameless. One example is the account of the revitalization of the aqueduct of Valens in Constantinople in 765, which, after a long period of disuse, required the requisitioning of a major workforce. As described in the early ninth‐century *Chronicle* of Theophanes, Emperor Constantine V

collected artisans from different places and brought from Asia and Pontos 1,000 masons and 200 plasterers, from Hellas and the islands 500 clay‐workers, and from Thrace itself 5,000 labourers and 200 brickmakers. He set taskmasters over them including one of the patricians. When the work had thus been completed, water flowed into the City. 30

### Mobility

This is where the concept of ‘mobility’ comes in. It widens the view beyond migration, which, as has already been noted, is usually understood in a broader demographic and geographic sense to involve the movement of large groups of people over long distances, often across political boundaries. Employing the concept of ‘mobility’ allows us to widen our perspective and to include a much wider range of people within our purview, whether they moved as individuals, families, clans or in smaller or larger groups, and whether they moved within a region, across wider distances or even beyond the empire. It also shifts the emphasis to the agency of those who move, rather than assuming that they are passive victims of circumstance, warfare or imperial policy. If such mobility occurs voluntarily, the agency of the actors is clear. But even if mobility is forced, the actors make choices, along the route and at their new destination, in how they comport themselves, with whom they seek contact, and how they attempt to manage in dangerous or disadvantageous situations.

The term ‘mobility’ has a further advantage: it allows us to imagine movement not only in geographical terms, from one point on the map to another, but can also be pictured as vertical mobility across social strata. Indeed, there is an intrinsic relation between the two: upward social mobility often requires geographical mobility.

If we wish to study people who are moving, we cannot neglect these social aspects. Here we can benefit from recent trends in modern migration studies that bring into focus not just the fact of movement or displacement from one location to another, but seek to understand the agency of individuals on the move as they continue to interact with their social networks in their locations of origin while building new networks at their destinations. In fact, the latter is often an extension of the former, as people who have recently arrived in a new place depend on support systems for the exchange of information and a helping hand, and these are most readily offered by the people to whom they are already connected and share the same language, i.e. migrants of the same background. The emphasis on personal agency in conjunction with mobility thus invites a closer look at microstructures, i.e. groups defined by a common origin, shared language, joint profession, or similar goals. 31

## Part Two: who gets to tell the story?

The written sources shape our perception of migration events, and thus require further scrutiny in their own right. Who gets to tell the story ultimately determines whose story is being told, in what literary context it is recounted, and what literary devices the authors employ. The authors choose what kind of movement and what actors they wish to present. They include these events in a larger narrative context that in itself gives significance and meaning to their chosen topic. And they harness literary tools, most prominently metaphors or adjectives, in order to achieve a certain effect. This is true especially when the author’s voice makes itself known, for example in a historical account or a saint’s *Life*. But even texts that are of a supposedly ‘neutral’ character, such as archival documents or legal writing, display these features of authorial choice.

What is of particular interest to the present argument is the scale of the mobility event – whether it concerns individuals, smaller groups such as families or clans, or larger groups such as people with a common ethnic, religious or regional origin – and its correlation to the other two aspects, literary context and literary devices. As long as the study of mobility and migration in Byzantium is still in the early stages and the full range of evidence is yet to be assembled to lay the groundwork for further study, paying attention to the literary constructedness of our sources is essential if we do not wish to fall into a ‘positivist’ trap. Such an approach not only helps us to gain a greater awareness that what we may be tempted to treat as historical ‘events’ is in fact the result of authorial interventions, but also goes a long way to explain the gaps in our knowledge and why there is so much of interest to the historian of mobility and migrations that our authors do not report. How did the regional or middle‐distance trade in ceramics that is attested through archaeological finds evolve? 32 What kinds of administrative protocols accompanied the forced resettlement of peoples from one region to another? Did individual women prepare for and conduct their movements differently from men?

Three examples can serve to illustrate how these issues are addressed in different literary contexts: in prescriptive texts emanating from the imperial centre of power; in historical narratives of larger movements by authors based in Constantinople; and by authors who focus on the stories of individuals.

### Prescriptive texts

Direct mention of mobility occurs in our period only in the very few texts that serve the military or fiscal interests of the central administration. Emperors were primarily interested in the mobility of their troops. Byzantium preserves an unusually large number of military handbooks, starting with the seventh century, most of them associated with emperors as authors: the *Strategikon* of Maurice, the *Taktikon* of Leo VI, plus three short military treatises by Leo’s son Constantine Porphyrogennetos. These all include advice for the preparation and execution of army movements on imperial soil and in enemy territory, in addition to ethnographic descriptions of enemy peoples and how to engage with them through diplomacy (including espionage) to avoid warfare, or – if that is not possible – instructions on how to adjust combat strategies depending on the enemy’s skills and equipment.

The *Taktikon* of Leo VI (r. 886–912) contains the well‐known and much‐discussed passage that lays out how his father Basil I had attempted to control the Slavs who had moved into the empire from north of the Danube:

Our father Basil, *autokrator* of the Rhomaioi, of pious memory, persuaded these peoples to abandon their old customs and, having made them ‘Greek’ (*graikôsas*), subjected them to rulers according to the Roman model, and having honoured them with baptism, he liberated them from submission to their own rulers and taught them to take part in warfare against the nations that fight against the Romans. In this way, he arranged these things carefully. As a result he caused the Rhomaioi to feel relaxed after the frequent uprisings by the Slavs in the past and the many upheavals and wars they had suffered from them in ancient times. 33

If there was ever an imperial integration and assimilation policy imposed on in‐migrants, these were the steps: abandonment of old customs, imposition of the Roman political system, integration in the ecclesiastical structure through baptism, and recruitment for military service – with the cumulative result of ‘Romanizing’ the Slavs, as John Haldon notes. 34 The verb *graikôsas* is an interesting choice of terms. After all, the designation of ‘Byzantium’ for the Christian medieval culture centred on Constantinople is anachronistic in its reference to its predecessor, the ancient city of Byzantion. It was coined at the end of the sixteenth century by Humanist scholars who were more interested in classical antiquity and its traces than in the medieval culture that followed it. The Byzantines called themselves ‘Rhomaioi’, emphasizing the fact that they represented the only unbroken continuation of the Roman imperial tradition, even while their language was Greek. They commonly referred to their language as ‘hellenic’, based on the ancient Greek adjective. The use in this passage of a derivative from the Latin *graecus* to denote the desired transformation of the new settlers (*graikosas*, a rare word) is thus particularly striking and has generated much comment. In the present context, as Johannes Koder has shown, it means both an induction into the Greek language as well as an adaptation to Roman/Byzantine culture. 35

Imperial interests were also behind the few passages in a legal work probably of the mid‐eighth century, the so‐called Farmers’ Law (*Nomos Georgikos*), which encourage the cultivation of land that had been abandoned by previous owners. The aim was to ensure the presence of tax payers and military recruits. ‘When a farmer who does not have the means to work his own land takes to flight and goes abroad, the yield of the land should be enjoyed by those who are taxed by the fiscal office …’ 36 This short statement merely hints at a wider issue that was also evident in the story, mentioned above, of the desperate farmer who contemplated flight after his ox had died: the inability of many farmers to pay the requisite taxes forced them to abandon their plots, which were then taken over by wealthier owners who thus further enlarged their properties. This development marks the early stages in the formation of an increasingly powerful landholding aristocracy. 37

These texts – one military, the other legal – were written with a prescriptive intent and in bland bureaucratese, addressing nameless imperial subjects. They represent the view from the centre of power, without particular regard for those who were affected by these imperial interventions.

### Historical narratives

When chroniclers or authors of larger historical narratives mention mobility or migration, they tend to report the movement of large groups of people. They often report imperially ordered population transfers from one area of the empire to another, for the purpose of resettling areas that had been depopulated as a result of warfare, or of removing inhabitants from exposed frontier zones. But these accounts are never neutral and often intend to convey a message beyond their ostensible topic.

A fine example is the chronicler Theophanes, dubbed ‘the Confessor’ because he was tortured during Iconoclasm. He relates with palpable disgust the forced settlement in 809/10 of large numbers of people from Asia Minor to the *Sklaviniai* at the Byzantine border regions in the Balkans, including the Peloponnese and Thrace.

In this year, after [having inflicted] ungodly punishments with the intention of impoverishing the troops [*strateumata*] in every way, Nikephoros displaced Christian people from all the *themata* and commanded that they be transferred to the *Sklaviniai*, and also that their property be sold. This action was no less grievous than captivity: many [of them] out of insanity engaged in blasphemies against God and implored to be attacked by the enemy, while others lamented at the tombs of their ancestors and extolled the bliss of the dead; there were also those who killed themselves by hanging in order to be spared from these tribulations. At the same time, since their possessions were too heavy to move [*dyskineta*], they were unable to carry them along and witnessed the loss of property acquired by the hard work of their parents. 38

This is a heart‐wrenching account, to be sure. But it is not told in a neutral context. Theophanes brings up this horrific tale amidst his complaints against the iconoclast emperor Nikephoros I, as the first in a list of his evil deeds that also includes harsher taxation and the oppression of the poor. In other words, this is a story told by an outside observer who instrumentalizes the migration episode for his own polemical purposes.

### Stories of individuals

For stories about mobility that focus on the fate of individuals, rather than large groups, we have to turn to hagiography, 39 or to ego‐accounts, i.e. those texts where authors speak in the first person, such as the poem by Mauropous mentioned above. Hagiographical tales of holy men and holy women are always predicated on rupture with their original surroundings in search of a higher goal. Indeed, the narratives of their lives are usually construed as a spiritual journey until they reach their final destiny in death and their relics find their destination in the tomb where their cult is celebrated. 40 Such narratives gain dramatic tension when historical circumstances re‐enforce the migratory aspect.

One example may suffice, the *Life of St Luke of Steiris*, the eponymous founder of the monastery of Hosios Loukas near Delphi. 41 Originally from Phocis, his life is a combination of voluntary departures for the pursuit of spiritual advancement and forced departures in search of safety from raids by slave traders, Bulgarians and Arabs. His movements, though frequent, do not cover great distances as they occur largely on both sides of the Gulf of Corinth. The narrative foil is provided by movements on a much larger scale, in the form of two travelling monks on pilgrimage from Rome to Jerusalem who turn up at key moments in Luke’s life. While the mobility of these anonymous pilgrims is depicted as serving their spiritual advancement, the hagiographer does not accord any spiritual value to Luke’s dramatic experiences of displacement, but merely records them without further comment.

While there are numerous hagiographical accounts that focus on the movement of their subjects, ego‐documents of individuals who represent themselves as having experienced mobility are rare in the period prior to 1000. Travel is seen as perilous and burdensome and is usually undertaken as a professional obligation or under duress, forced by the cruel fate of captivity. It is certainly not valued as adding insight and experience. After the sixth century, when the Alexandrian trader Cosmas Indicopleustes (‘the Traveller to India’) described India and Ceylon as well as the kingdom of Axum, there is a distinct lack of interest among Byzantine authors of subsequent centuries in creating accounts of their travels. Yet travel they did, as diplomats in the service of church and empire or as traders, all of them of a sufficiently high social standing to command the requisite literary skills for such an undertaking. Anthony Kaldellis explains this remarkable silence about regions beyond the Empire with the new self‐congratulatory, inward‐looking Christian‐imperial stance that became a point of pride among the authors of the middle Byzantine period. 42 This begins to change from the late eleventh century, when Byzantine authors compose texts in an autobiographical mode that reflect on their past lives. Many of them had experienced travel and displacement first hand. It was precisely those experiences that added drama to their lives and made them worth recounting. 43

Especially telling is the outlook of those who report the fate of enforced displacement they suffered as a result of warfare. 44 The earliest such account comes from John Kaminiates, a prominent clergyman, who experienced the capture of Thessaloniki by the Arabs in 904. His life was spared and he was taken to Tripoli and eventually to Tarsos, where he was held for ransom along with other prisoners of war. His recollections present themselves as a letter written in response to a request by another prisoner of war, with whom he had crossed paths for a brief moment.

You sought in your letter to learn the way in which it had come about that we dwell in captivity, having been delivered into the hands of barbarians, how we exchanged a foreign land for our own country, *where we hail from and what sort of place it is*. 45

Here, too, captivity and displacement are only part of the story. Gregory of Cappadocia, John’s addressee, had apparently seized the opportunity of meeting a man from Thessaloniki to satisfy his curiosity about that distant place. As a result, the first fifteen out of a total of seventy‐eight chapters contain a detailed description of the city, its buildings and its inhabitants, before turning to the capture and its aftermath. 46

Indeed, John Kaminiates may be regarded as belonging to a long line of authors who report historical events not only because they experienced them or played a role in them (a line that reaches back to Procopius of Caesarea in the sixth century and his stylistic models Herodotus and Thucydides in ancient Greece), but who know that they have important, edifying or entertaining stories to tell based on their own mobility, even if it was enforced and involuntary. 47

As these examples of historical narratives, hagiography or ego‐accounts show, telling the story of mobility and migration is thus always more than a simple act of recording. As we approach these texts, we must be wary of cherry‐picking them for details of historical events, valuable though this may be. We must always be attentive to the fact that the authors told their stories for a specific purpose. More often than not, they are presenting us not with cherries ripe for the picking, but – to stay with the orchard metaphor – with carefully cultivated fruit of their own making. We must never forget that those who chose to tell the stories of mobility and migration did so for reasons very much their own.

## Conclusion: mobility and scale in Byzantine texts

Taking a lead from Stouraitis’ diagram of types of migration in Table 1 above, it may be helpful to visualize in a new table which authors report mobility on what scale and in which literary mode. The diagram below attempts to illustrate this (Table 2). It distinguishes between migration beyond Byzantium and intra‐Byzantine mobility, and notes whether this was a peaceful or violent event. A further subdivision is made according to the number of people involved.

**Table 2:** Mobility and migration in Byzantine texts

	*In and Out of Byzantium*		*Within Byzantium*	
	*Peaceful*	*Violent*	*Peaceful*	*Violent*
*Large groups of people*	Historical narratives (e.g. in‐migration of Armenians)	Historical narratives	Scattered short references (e.g. pastoral nomads)	Historical narratives (e.g. forced settlement)
*Individuals and families*	Ego‐accounts of ambassadors	Ego‐accounts of captives (Niketas Choniates, John Kaminiates)	Scattered short references	Biographical narratives (hagiography);Ego‐accounts, esp. letters of exiles
	Ego‐accounts of success stories (Gregory Pakourianos, Eustathios Boilas)			

What is made abundantly clear (if indeed we needed confirmation) is that movements, including migrations, of large groups of people accompanied by violence attracted the greatest attention in the contemporary sources. These are usually reported in historical accounts written by narrators who assume the stance of a distant observer. As these sources form the backbone of historical scholarship, these are the types of movement that are best known today. Texts written in the first person (ego‐accounts) are narrower in scope, but offer more vivid detail. This is true even if the movement was peaceful, but becomes emotionally compelling when the movement involved violence. The focus lies on the personal experience of the narrator and, especially in the instances of forcible movement, that of his family members or close companions.

The kind of mobility that attracted the least attention from Byzantine authors (and hence the modern scholars who depend on them) is peaceful movement, whether by large groups or by individuals. It is usually only reported in passing, and not the focus of the text: entertainers who travel from city to city, women who sell their vegetables at market, young men from the provinces who pursue their education in Constantinople, itinerant crews of craftsmen who ply their trade in different locations. The diagram thus also draws attention to the gaps in our knowledge. Yet it is these occurrences that are testimony to Byzantium, not as a periodic victim of violent incursions, but as a society whose vibrancy and functioning depended on mobility.

## Footnotes

^1^
J. Preiser‐Kapeller, L. Reinfandt and Y. Stouraitis (eds), *Migration Histories of the Medieval Afroeurasian Transition Zone. Aspects of Mobility between Africa, Asia and Europe, 300–1500 c.e*., Studies in Global Migration History 13 (Leiden and Boston, 2020, open access); L. Brubaker, R. Darley and D. Reynolds (eds), *Global Byzantium. Papers from the Fiftieth Spring Symposium of Byzantine Studies* (London, 2022).

^2^
C. Holmes and N. Standen, ‘Introduction: Towards a Global Middle Ages’, *Past and Present*, Supplement 13 (2018), pp. 1–44, p. 28, with reference to the contribution by Standen and White in the same volume.

^3^
F. Trivellato, *The Familiarity of Strangers. The Sephardic Diaspora, Livorno, and Cross‐Cultural Trade in the Early Modern Period* (New Haven, 2009), quotations at p. 7 and p. 2.

^4^
E.A. Lambourn, *Abraham’s Luggage. A Social Life of Things in the Medieval Indian Ocean World* (Cambridge, 2018).

^5^
A. Eastmond, *Tamta’s World. The Life and Encounters of a Medieval Noblewoman from the Middle East to Mongolia* (Cambridge, 2017).

^6^
J.‐P. Ghobrial, ‘Moving Stories and What They Tell Us: Early Modern Mobility between Microhistory and Global History’, *Past and Present*, Supplement 14 (2019), pp. 243–80; see also his ‘Introduction: Seeing the World like a Micro‐Historian’, *ibid*., pp. 1–22.

^7^
Figures given by D. Stathakopoulos, ‘Population, Demography, and Disease’, in E. Jeffreys, with J. Haldon and R. Cormack (eds), *The Oxford Handbook of Byzantine Studies* (Oxford, 2008), pp. 307–16, at p. 310. See especially J. Koder, *Der Lebensraum der Byzantiner. Historisch‐geographischer Abriss ihres mittelalterlichen Staates im östlichen Mittelmeerraum*, Byzantinische Geschichtsschreiber, Ergänzungsband 1 (Graz etc., 1984; repr. with expanded bibliography Vienna, 2001).

^8^
H. Ditten, *Ethnische Verschiebungen zwischen der Balkanhalbinsel und Kleinasien vom Ende des 6. bis zur zweiten Hälfte des 9. Jahrhunderts* (Berlin, 1993); H. Ahrweiler and A. Laiou (eds), *Studies on the Internal Diaspora of the Byzantine Empire* (Washington, DC, 1998); M. Balard and A. Ducellier (eds), *Migrations et diasporas méditerranéennes (Xe–XVIe siècles). Actes du colloque de Conques (octobre 1999)*, Byzantina Sorbonensia 19 (Paris, 2002); Y. Stouraitis, ‘Migrating in the Medieval East Roman World, ca. 600–1204’, in Preiser‐Kapeller, Reinfandt and Stouraitis (eds), *Migration Histories of the Medieval Afroeurasian Transition Zone*, pp. 141–75; C. Rapp, ‘Zwangsmigration in Byzanz. Kurzer Überblick mit einer Fallstudie aus dem 11. Jahrhundert’, in Th. Ertl (ed.), *Erzwungene Exile. Umsiedlung und Vertreibung in der Vormoderne (500–850)* (Frankfurt a.M. and New York, 2017), pp. 59–79.

^9^
‘Mobility, Microstructures and Personal Agency’, FWF Project Z 288‐G25 (Wittgenstein Award for Claudia Rapp), rapp.univie.ac.at (last access 14 January 2023).

^10^
See, for example, C. Rapp and Y. Stouraitis (eds), *Microstructures and Mobility in Byzantium* (Vienna and Göttingen, forthoming in print and open access).

^11^
C. Rapp *et al*., *Mobility and Migration in Byzantium: A Sourcebook* (Vienna and Göttingen, 2023). Co‐authors are Matthew Kinloch, Dirk Krausmüller, Ekaterini Mitsiou, Ilias Nesseris, Giulia Rossetto, Christodoulos Papavarnavas, Johannes Preiser‐Kapeller, Rustam Shukurov and Grigori Simeonov.

^12^
John Mauropous, *Poems*, nos. 47–8: *The Poems of Christopher of Mytilene and John Mauropous*, ed. F. Bernard and C. Livanos, Dumbarton Oaks Medieval Library 50 (Cambridge and London, 2018), pp. 410–16 (text and trans., modified by D. Krausmüller).

^13^
Demetrios Chomatenos, *Various Works*, no. 50: *Demetrii Chomateni Ponemata Diaphora*, ed. G. Prinzing, Corpus Fontium Historiae Byzantinae 38 (Berlin and New York, 2002), pp. 183–4 (text), cf. pp. 125*–126*. My translation.

^14^
Niketas, *Life of Saint Philaretos*, c. 3: *The Life of Philaretos the Merciful Written by his Grandson Niketas*, ed. L. Rydén (Uppsala, 2002), pp. 64–7.

^15^
Eustathios Boilas, *Testament*, in *Cinq études sur le XI siècle byzantin*, ed. P. Lemerle (Paris, 1977), pp. 20–9 (text); S.J. Vryonis, ‘The Will of a Provincial Magnate, Eustathius Boilas (1059)’, *Dumbarton Oaks Papers* 11 (1957), pp. 263–77, at pp. 264–72 (trans.). Rapp, ‘Zwangsmigration in Byzanz’ with further references.

^16^
Gregorios Pakourianos, *Typikon*, c. 24, in ‘Le typikon du sébaste Grégoire Pakourianos’, ed. P. Gautier, *Revue des études byzantines* 42 (1984), pp. 5–145, at p. 105 (text); ‘Typikon of Gregory Pakourianos for the Monastery of the Mother of God Petritzomitissa in Backovo’, ed. R. Jordan, in J. Thomas and A. Constantinides Hero (eds), *Byzantine Monastic Foundation Documents* (Washington, DC, 2000), pp. 507–63, at p. 547 (trans.). The document was written in Greek, Georgian and Armenian, at Pakourianos’ demand.

^17^

*Euchologium Barberini*, no. 192: *L’euchologio Barberini gr. 336*, ed. S. Parenti and E. Velkovska, 2nd edn (Rome, 2000), pp. 189–90 (text), p. 349 (trans.) (repeated in 3rd edn Omsk, 2011, p. 402). This prayer is also preserved, with slight variations, in several later manuscipts. My translation. I thank Elisabeth Schiffer for her valuable comments on this text.

^18^
F.K. Haarer, ‘Writing Histories of Byzantium: The Historiography of Byzantine History’, in L. James (ed.), *A Companion to Byzantium* (Chicester, 2010), pp. 8–21, demonstrates how the notions of ‘decline’ and ‘crisis’ have long dominated scholarly engagement with Byzantium.

^19^
J. Pahlitzsch, ‘Byzanz’, in M. Borgolte (ed.), *Migrationen im Mittelalter. Ein Handbuch* (Berlin, 2014), pp. 93–106.

^20^
P. Charanis, *The Armenians in the Byzantine Empire* (Lisbon, 1963); and his collected essays in P. Charanis, *Studies on the Demography of the Byzantine Empire* (London, 1972); J. Preiser‐Kapeller, ‘Aristocrats, Mercenaries, Clergymen and Refugees: Deliberate and Forced Mobility of Armenians in the Early Medieval Mediterranean and Near East (6th to 11th Century)’‚ in Preiser‐Kapeller, Reinfandt and Stouraitis (eds), *Migration Histories of the Medieval Afroeurasian Transition Zone*, pp. 327–84.

^21^
Theophanes Continuatus, *Life of Basil*, c. 2: *Chronographiae quae Theophanis continuati nomine fertur liber quo Vita Basilii imperatoris amplectitur*, ed. I. Sevcenko, Corpus fontium historiae byzantinae 42, ser. Berol. (Berlin and Boston, 2011), pp. 10–15 (text and trans.), quotation on p. 15.

^22^
T. Greenwood, ‘Basil I, Constantine VII and Armenian Literary Tradition in Byzantium’, in T. Shawcross and I. Toth (eds), *Reading in the Byzantine Empire and Beyond* (Cambridge, 2018), pp. 447–66.

^23^
In view of this, Anthony Kaldellis has taken issue with the tendency of modern scholars to assume that Armenian ‘identity’ (or ‘nationality’ or ‘blood’) was passed on as a biological feature, labelling this line of reasoning ‘The Armenian Fallacy’. A. Kaldellis, *Romanland. Ethnicity and Empire in Byzantium* (Cambridge, MA and London, 2019), pp. 155–95.

^24^
N. Garsoian, *The Paulician Heresy. A Study of the Origin and Development of Paulicianism in Armenia and the Eastern Provinces of the Byzantine Empire* (The Hague, 1967; repr. Berlin, 2011); C. Dixon, *The Paulicians. Heresy, Persecution and Warfare on the Byzantine Frontier, c.750–880*, The Medieval Mediterranean 132 (Leiden and Boston, 2022).

^25^
Stouraitis, ‘Migrating in the Medieval East Roman World’, p. 143.

^26^
P. Melichar, *Empresses of Late Byzantium: Foreign Brides, Mediators, Pious Women* (Berlin, 2019) is a model study for later centuries.

^27^
Ch. Makrypoulias, ‘Ranks to Riches: The Social Mobility of Middle Byzantine Infantry’, in Rapp and Stouraitis (eds), *Microstructures and Mobility*.

^28^
See for example A. Cutler, ‘Significant Gifts. Patterns of Exchange in Late Antique, Byzantine, and Early Islamic Diplomacy’, *Journal of Medieval and Early Modern Studies* 38.1 (2008), pp. 79–101.

^29^
For general context, see Preiser‐Kapeller, Reinfandt and Stouraitis (eds), *Migration Histories of the Medieval Afroeurasian Transition Zone*.

^30^
Theophanes Confessor, *Chronography* A. M. 6258, ed. C. de Boor, 2 vols (Leipzig, 1883–5), vol. 1, p. 440; *The Chronicle of Theophanes Confessor: Byzantine and Near Eastern History a.d. 284–813*, ed. C. Mango and R. Scott (Oxford, 1997), p. 608 (trans. modified by Dirk Krausmüller).

^31^
For an overview, see C. Harzig and D. Hoerder, with D. Gabaccia, *What is Migration History?* (Cambridge, 2009); D. Hoerder, *Cultures in Contact. World Migrations in the Second Millennium* (Durham and London, 2002); S. Hahn, *Historische Migrationsforschung* (Frankfurt a.M., 2012).

^32^
F. Liard, ‘Pottery Traditions as Indicators of Interactions, Connectivity and Microstructures in Byzantium’, in Rapp and Stouraitis (eds), *Microstructures and Mobility*.

^33^
Leo, *Taktika*, Constitution, 18.93–5: *The Taktika of Leo VI. Text, Translation, and Commentary*, ed. G. Dennis, Corpus Fontium Historiae Byzantinae 49 (Washington, DC, 2014), pp. 470–1 (text and trans., modified). See also J. Koder, ‘On the Slavic Immigration into the Byzantine Balkans’, in Preiser‐Kapeller, Reinfandt and Stouraitis (eds), *Migration Histories of the Medieval Afroeurasian Transition Zone*, pp. 81–100.

^34^
J. Haldon, *A Critical Commentary on the* Taktika *of Leo VI* (Washington, DC, 2014), pp. 350–1.

^35^
J. Koder, ‘Anmerkungen zu ΓΡΑΙΚΟΩ’, *Byzantina* 21 (2000), pp. 199–202, at p. 201 speaks of ‘griechische Sprachvermittlung’ and ‘Kulturanpassung’.

^36^

*Nomos Georgikos*, c. 18 (see also cc. 11, 13, 14, 21): *Nomos Georgikos. Das byzantinische Landwirtschaftsgesetz. Überlegungen zur inhaltlichen und zeitlichen Einordnung. Deutsche Übersetzung*, ed. J. Koder, Wiener Byzantinistische Studien 32 (Vienna, 2020), pp. 49–50 (see also pp. 47, 47–8, 48, 51) (text).

^37^
A. Harvey, *Economic Expansion in the Byzantine Empire, 900–1200* (Cambridge, 2011), esp. p. 34.

^38^
Theophanes, *Chronography* AM 6302, ed. de Boor, vol. 1, pp. 486–7 (text); *The Chronicle of Theophanes Confessor*, ed. Mango and Scott, pp. 667–8 (trans. modified by Christodoulos Papavarnavas).

^39^
For hagiographical narratives of mobility (or its absence), see the contribution of Christodoulos Papavarnavas to this volume.

^40^
On the hagiographical topos of holy men escaping from fame, see, *inter alia*, A. Kazhdan, ‘Hagiographical Notes’, *Byzantinische Zeitschrift* 78 (1985), pp. 49–55, at pp. 50–3.

^41^

*Life and Mircales of Saint Luke of Steiris*, ed. C.L. Connor (Brookline, 1994) (text and trans.).

^42^
A. Kaldellis, *Ethnography after Antiquity. Foreign Lands and Peoples in Byzantine Literature* (Philadelphia, 2013).

^43^
M. Hinterberger, *Die byzantinische Autobiographie*, Ph.D. thesis, University of Vienna (1996), p. 394 notes that most autobiographical authors could look back on a life that was rich in changes of location.

^44^
Grigori Simeonov treats the fate of those who were displaced as a result of warfare in detail in his contribution to this volume. For the significance of texts written by authors who experienced traumatic suffering, see now A.J. Goldwyn, *Witness Literature in Byzantium. Narrating Slaves, Prisoners, and Refugees* (Cham, 2021).

^45^
John Kaminiates, *The Capture of Thessalonica*, c. 2: *Ioannis Caminiatae De expugnatione Thessalonicae*, ed. G. Böhlig, Corpus Fontium Historiae Byzantinae 4 (Berlin and New York, 1973), p. 4 (text); *John Kaminiates, The Capture of Thessaloniki. Translation, Introduction and Notes*, ed. D. Frendo and A. Fotiou, Byzantina Australiensia 12 (Perth, 2000), p. 5 (trans.).

^46^
There are other, psychological and literary, reasons that can explain the excursus on Thessaloniki: reminiscing about his city in times of peace and prosperity may have been welcome to John as an opportunity to remember happier days, and offers the audience a foil for the brutal descriptions that follow. Giving a systematic introduction to the city and its infrastructure also facilitates the understanding of the different locations that are mentioned in the subsequent narration of the capture of the city and its inhabitants.

^47^
See also C. Messis, ‘La mémoire du “je” souffrant. Construire et écrire la mémoire personelle dans les récits de captivité’, in P. Odorico, P.A. Agapitos, M. Hinterberger (eds), *L’écriture de la mémoire. La littérarité de l’historiographie. Actes du IIIe colloque international philologique ‘EPMHNEIA’, Nicosie, 6‐7‐8 mai 2004 organisé par l’E.H.S.S. et l’Université de Chypre* (Paris, 2006), pp. 107–46.

